# Vibration Influences Haptic Perception of Surface Compliance During Walking

**DOI:** 10.1371/journal.pone.0017697

**Published:** 2011-03-25

**Authors:** Yon Visell, Bruno L. Giordano, Guillaume Millet, Jeremy R. Cooperstock

**Affiliations:** 1 Centre for Intelligent Machines and CIRMMT, McGill University, Montreal, Quebec, Canada; 2 Department of Music Research and CIRMMT, McGill University, Montreal, Quebec, Canada; University of Sydney, Australia

## Abstract

**Background:**

The haptic perception of ground compliance is used for stable regulation of dynamic posture and the control of locomotion in diverse natural environments. Although rarely investigated in relation to walking, vibrotactile sensory channels are known to be active in the discrimination of material properties of objects and surfaces through touch. This study investigated how the perception of ground surface compliance is altered by plantar vibration feedback.

**Methodology/Principal Findings:**

Subjects walked in shoes over a rigid floor plate that provided plantar vibration feedback, and responded indicating how compliant it felt, either in subjective magnitude or via pairwise comparisons. In one experiment, the compliance of the floor plate was also varied. Results showed that perceived compliance of the plate increased monotonically with vibration feedback intensity, and depended to a lesser extent on the temporal or frequency distribution of the feedback. When both plate stiffness (inverse compliance) and vibration amplitude were manipulated, the effect persisted, with both factors contributing to compliance perception. A significant influence of vibration was observed even for amplitudes close to psychophysical detection thresholds.

**Conclusions/Significance:**

These findings reveal that vibrotactile sensory channels are highly salient to the perception of surface compliance, and suggest that correlations between vibrotactile sensory information and motor activity may be of broader significance for the control of human locomotion than has been previously acknowledged.

## Introduction

The goal of this study is to measure empirically the role played by vibrotactile sensory information in the perception of ground surfaces during walking. To this end, we focused on a basic property of walking surfaces that is highly salient to locomotion—their mechanical compliance [1], [2]. We investigated what influence, if any, vibration feedback to the plantar soles may have on the perception of ground surface compliance during walking.

The perception of ground surfaces is instrumental to enabling us to move easily on foot in diverse natural environments. Human locomotor movements are adapted when stepping onto, off of, or moving over soft, irregular, or slippery surfaces in ways that minimize metabolic costs, reduce impact forces, or stabilize vertical center of mass [1]–[7]. Compliant ground surfaces, such as sand or soggy grass, perturb locomotion by degrading proprioceptive cues that are acquired via ground contact and by mechanical perturbations due to the compression of material underfoot. Walkers automatically modulate their gait pattern and biomechanics to compensate for such changes in compliance [2].

When haptic sensation in the feet is impaired, as a result of a disease such as diabetes, or through local anesthesia, it can have detrimental effects on locomotion [8]–[11]. However, knowledge about the influence of different sources of haptic sensory information, such as plantar force or vibromechanical stimuli, on the control of walking is incomplete.

Haptic compliance perception involves discerning the deformability of objects touched with the hand, or of surfaces felt underfoot. Compliance, the inverse of stiffness, is the ratio between displacement and applied force, 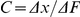, and is related to the intrinsic material property of elasticity. Most prior research has investigated compliance perception via manual touch [12]–[19], but the haptic perceptual system is also able to discriminate walking surfaces of different elasticity [20], [21]. Sensitivity is highest when there is direct contact between the surface of the skin and a deformable object. In this setting, cutaneous tactile cues predominate [17]. Conversely, when touch is mediated by a rigid link, such as a stick or a stiff shoe sole, cutaneous force cues are combined with proprioceptive information to form compliance estimates [15], [16], [19], [22]. If cutaneous information is blocked entirely, performance is greatly degraded [16].

While we are not aware of any prior investigation of effects of vibrotactile sensory information on compliance perception, it is well established that high-frequency mechanical vibrations generated during interaction with surfaces via manually tapping or scraping with a probe, or scanning with a finger, can influence the perception of properties such as hardness and texture [23]–[28]. For example, amplifying vibrations generated during manual surface scanning, or imposing sinusoidal vibrations, increases perceived surface roughness [29]. Vibrations produced during frictional sliding are indicative of movement [30], and could contribute to compliance perception. On this basis, it could be hypothesized that an amplification of plantar vibration intensity would lead to an increase in the magnitude of compliance estimates, because displacement and compliance are proportional.

Mechanical signals generated during walking on natural ground surfaces constitute a rich source of haptic sensory information [31]–[33]. The compression of many heterogeneous materials (e.g., wood, snow, gravel) results in inelastic, unrecoverable deformations with energy distributed over a broad frequency band [31]. The pattern of these vibrations is highly correlated with material displacement [34], [35], so it is natural to consider them as potential displacement cues. Giordano et al. found that walkers are able to distinguish between the feel of porous and solid ground surfaces, or rock gravel surfaces of different grades, when walking in shoes [36]. When plantar cutaneous input was masked by mechanical vibrations, in the form of synthesized pseudo-random noise (frequency distribution: 50 Hz to 1 kHz), performance was impaired, suggesting that vibrotaction played a significant role. (Further analysis is provided in: Giordano B, Visell Y, Cooperstock JR, Yao HY, Hayward V, and McAdams S (2010) Audiohaptic identification of ground materials during walking, Submitted.)

Relatively few studies have investigated haptic perception with the feet. However, the foot is serially homologous to the hand, and is highly evolved as a sensory instrument. Its perceptual-motor abilities are involved in the regulation of posture and locomotion [37], and in the estimation of ground slipperiness and slant [38]–[40]. The sensory physiology of the plantar sole is highly developed, with the same type of mechanoreceptor populations as are present in the hand: the fast-adapting (FA) type I and II and slow-adapting (SA) type I and II receptors [41], [42]. The sole is highly sensitive to vibration, with FA receptors comprising about 70% of the cutaneous population. Low-frequency forces are sensed by SA receptors [41], and by Golgi organs, muscle spindles, and joint capsule receptors in the muscles, tendons, and joints.

Vibromechanical stimulation of the plantar sole affects both cutaneous receptors and deeper foot and ankle proprioceptors. Such stimuli can result in real or illusory postural effects resembling those due to an increase in local pressure at the same location of the foot sole [43]–[46]. This could be taken to suggest that amplifying plantar vibration may, by increasing perceived forces, decrease ground compliance estimates—contrary to what is suggested by foot-ground mechanical considerations. However, studies of this type have generally been conducted while subjects stood in place, whereas haptic compliance perception always requires movement. In other experiments on vibration stimulation of the leg muscles or tendons, different effects have been observed to accompany stimulation provided during stance than those induced when it is provided during locomotion. In the former case, it induces whole-body postural tilts (attributed to illusory lengthening of the stimulated muscles), whereas during locomotion it results in modified stepping movements with little overall change in muscle coordination [47]–[49]. Courtine et al. argued that this reflects the fact that sensory inflow is processed depending on both the body segment where it arises and the performed task [48]. As a result, we questioned whether prior results on postural effects of plantar vibration would apply in our study, in which subjects were actively moving.

Our experiments evaluated the influence of vibrotactile information felt during stepping onto a floor surface on the perceived compliance of the latter. The above-referenced studies involved a diverse range of signal types (noise-like or natural textures, and sinusoidal stimuli), amplitudes, and temporal dependencies. Experiment 1 was designed to investigate effects of vibration feedback on perceived compliance, and to clarify their dependency on time- and frequency-domain stimulus properties. To further determine the extent to which vibrotactile sensory information is combined with cutaneous force and proprioceptive information in the perception of ground compliance, Experiment 2 measured the effect of plantar vibration on compliance perception via a novel apparatus that allowed both the mechanical stiffness of a floor plate and vibration feedback presented through it to be manipulated. Psychophysical amplitude detection thresholds for the stimuli were also measured in order to provide an indication of the relative intensity of the stimuli.

## Materials and Methods

### Ethics Statement

The experiments were conducted in accordance with McGill University ethics guidelines, and was reviewed and approved by the McGill Research Ethics Board in accordance with the requirements of the McGill University Policy on Ethical Conduct of Research involving Human Subjects and with the Tri-Council Policy Statement: Ethical Conduct For Research Involving Humans.

### General Methods

During the experiments, subjects crossed a short walkway incorporating an actuated floor plate that provided vibration feedback in response to forces exerted by the foot. The mechanical stiffness of the plate was manipulated in Experiment 2.

### Apparatus

**Figure 1:**
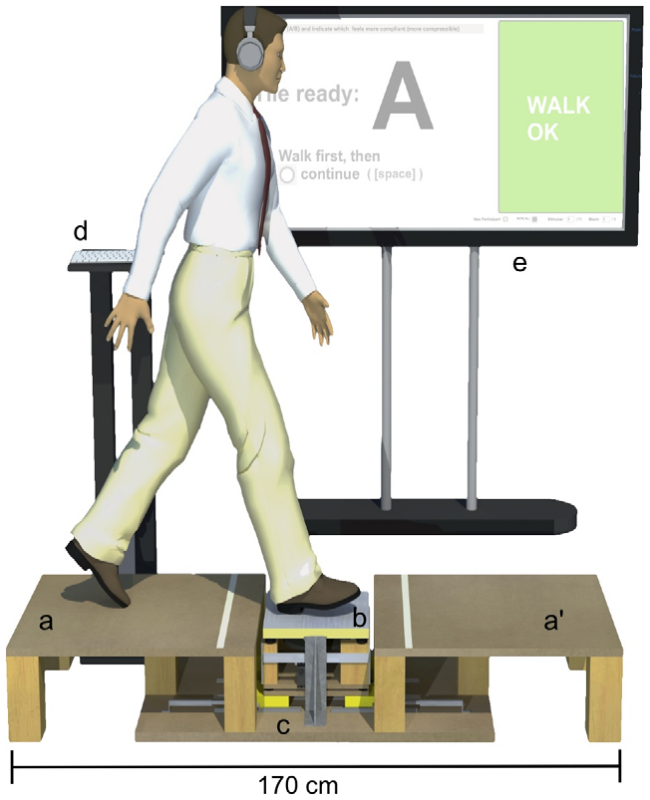
Apparatus for producing compliance and vibration stimuli. Subjects stepped from one side of the platform a onto the vibrating plate b, and onto the opposite platform a'. They then turned, stepped on b again, and returned to a. In Experiment 2, the plate also displaced up to 2 cm in the vertical direction, compressing a volume of EVA foam that was controlled by the linear servomechanism c, to produce the commanded compliance (see Fig. 3). Subjects entered their responses after each trial at the keyboard d and received instructions from the large-screen video monitor e.

The apparatus consisted of a short walking platform (Fig. 1) permitting subjects to take a single step onto a vibration-actuated floor plate. The plate was actuated by a Lorentz force inertial motor (Clark Synthesis model TST429) rigidly coupled to it from beneath. This plate was used to present walkers with vibration feedback and to present a specified mechanical stiffness to the walker, via a servo controlled mechanism (see Experiment 2).

To ensure that vibromechanical stimuli could be reproduced accurately across a wide range of frequencies, while assuring the stability of the plate under a human walker, we undertook an extensive redesign of our earlier apparatus [50], as fully described in reference [51]. Through measurements, we determined that the device was able to reproduce arbitrary vibrations accurately at forces of more than 40 N within a flat frequency band from 50 to 750 Hz. Within the range of amplitudes used here, vibrations could be presented with a nonlinear waveform distortion of less than 3% (mean absolute percent error, measured at 300 Hz). The device could sense static or transient loads of more than 1000 N supplied by a human foot. Analog data from the force sensors were conditioned, amplified, and digitized via a 16-bit acquisition card (National Instruments model USB-6218). Digital-to-analog conversion of the vibration signal was performed using a 24-bit, 48 kHz audio interface (Edirol model FA-101). The analog signal was then passed through a power amplifier driving the actuator. In order to assess the accurate reproduction of the vibration stimuli, they were independently recorded with a miniature accelerometer permanently attached to the underside of the plate.

### General Procedure

During the experiments, stimuli were presented via the plate as subjects stepped on it. They began on one side of the walkway (see Fig. 1), stepped onto the plate with their dominant foot, and proceeded to the opposite side, turned, stepped on the plate again using their dominant foot, returned to the first side, and entered their responses via a computer terminal. Before each experiment, subjects were instructed in the use of the apparatus and interface.

Both experiments took place in a structurally isolated, soundproofed room with a noise-floor rating of PNC20. Subjects wore foam earplugs with an NRR attenuation rating of 33 dB and wireless headphones playing pink noise at a volume sufficient to mask any sounds produced by the vibrating plate and the motors. The non-vibrating walking platforms were isolated from the actuators via cushioning material, eliminating the transmission of vibrations to users before stimulus presentation.

A steady walking pace was enforced via a 1 Hz metronome sound audible above the pink noise. The experiments were conducted at low light levels to allow subjects to focus on what they felt, but sufficient for the walkway to remain visible. However, subjects were asked to avoid looking down at the plate while walking on it, unless necessary to maintain equilibrium, and instead, were instructed to attend to one of the two static visual markers that were positioned at a height of 1.3 m (above foot level), and a distance 1 m from either end of the walkway.

Subjects were required to wear shoes in the experiment in order to avoid directing their attention to the surface properties of the plate. In order to standardize footwear in all experiments, only male subjects were recruited, with North American shoe size between 7 and 12. Each was given an identical model men's hard soled dress shoe in the appropriate size to wear. All subjects reported normal tactile sensation in the feet, with normal walking ability, and were naive with respect to the purpose of the study. They were presented with and signed informed consent forms at the beginning of the experiment and were paid ten dollars (CAD) per hour for their participation upon completion.

### Experiment 1

Experiment 1 was based on ratings of subjective compliance. We investigated the perceived compliance of a rigid plate augmented with nine different types of vibration feedback at two amplitude levels, as well as one condition in which no vibration feedback was provided. Subjects walked across each configuration of the plate, and rated its compliance on a continuous scale.

#### Recruitment

Twenty people participated in the experiment (mean age 24.5 years, STD = 6.9 years, average mass 70.9 kg, STD = 11.2 kg). None participated in any other study on compliance or vibration perception. Other details were as described under “General Methods”.

#### Stimuli

The stimuli consisted of several different types of vibration feedback and one no-vibration reference condition. The stiffness of the plate was held constant, and was set equal to 90 N/mm, the median stiffness value used in Experiment 2. The 18 vibration stimuli were generated by factorial combination of three parameters: amplitude scale 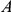 (0.5, 1.0), temporal waveform type 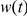 (Sinusoidal, White Noise, Textured Noise), and amplitude envelope 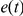 (Constant, Force-Proportional, Dynamic). Each stimulus can be described as an acceleration signal delivered from the plate, having the form 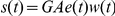, where 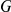 were stimulus-dependent peak gain factors.

**Figure 2:**
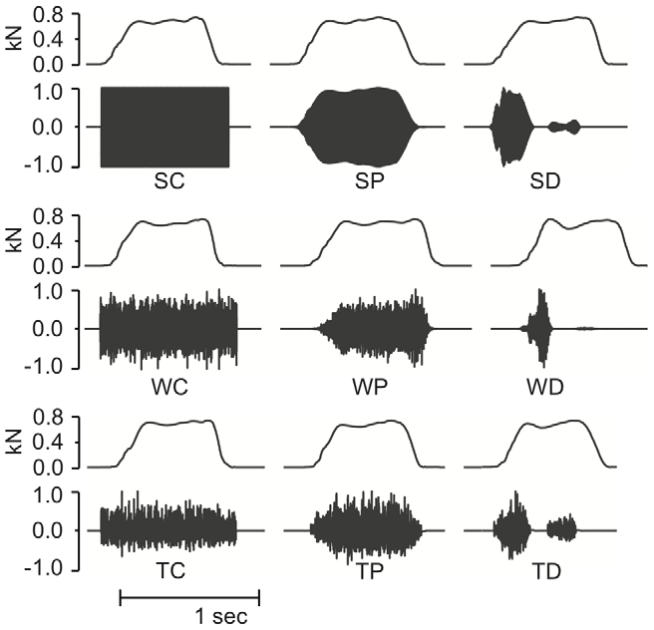
Vibration feedback stimuli. The thin lines (rows 1, 3, and 5) show force profiles from footsteps of one participant onto the plate, and the darker waveforms are the corresponding vibration feedback stimuli. Vibrations could be felt only during foot-plate contact. Stimuli are labeled with a 2-letter string, with the first encoding waveform type (S, W, T =  Sinusoidal, White noise, Textured noise), and the second encoding envelope type (C, P, D =  Constant, Proportional, Dynamic). The vibration amplitude range was normalized for display purposes.

The vibration signals for the nine stimuli resulting from combining the factors “amplitude envelope” and “waveform type” are shown in Fig. 2. Three different waveforms 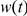 were used. The first was a sinusoid 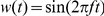 with frequency 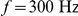. The second was a white noise that was band-limited by filtering to remove frequencies above 700 Hz and below 50 Hz. The third was a noise signal intended to resemble the texture felt when a porous material, such as gravel, is compressed. It was obtained by passing an impulsive noise source, consisting of a random impulse train, through a resonant filter. The impulses were identical in amplitude scale, and occurred at times 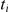 whose time intervals 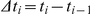 were sampled from a Poisson stochastic process; the intervals were distributed as 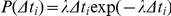. The mean event frequency was 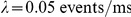. Each impulse was rendered as a 1 ms white noise burst beginning at 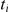. The impulse train was passed through a second-order infinite impulse response (IIR) bandpass filter with center frequency 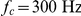 and bandwidth 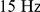. The resulting noise had a rough texture with most energy concentrated in a narrow frequency band at which FA type II mechanoreceptors in the foot sole are most sensitive.

The three different envelopes 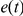 specified the amplitude profile of the vibration feedback in response to a footstep with normal force profile 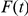. The first was a constant function 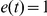, and the second was a linear force-proportional envelope 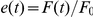, with inverse slope 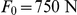. The third was a dynamic envelope 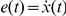 derived from an admittance-based simulation of a linear, compressible material, where 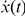 is the time-derivative of the virtual strain 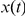 response to 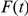, described by
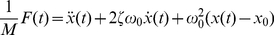
(1)The envelope 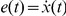 is obtained in real time as the output of a second-order digital IIR bandpass filter computed by solving (1) using the Laplace transform method and the bilinear transform [52], [53]. The filter input was 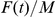 and the output was 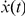. 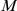 could be taken as the mass of a representative volume element, but here it is an arbitrary gain factor. We used frequency 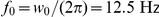, and set 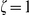 for critical damping. This yielded a characteristic envelope response time of 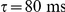.

As explained in reference [51], the combination of the dynamic envelope type with the textured noise waveform (i.e., stimulus TD) can be regarded as a simplified micromechanical model for the production of textured vibrations during the compression of a natural, heterogeneous material such as gravel, sand, or snow.

#### Stimulus intensity equalization

In a pilot study, we observed that the vibration feedback stimuli could significantly increase subjective compliance ratings, and that the effect depended primarily on the stimulus amplitude parameter. However, RMS signal energy and subjective stimulus intensity depended on amplitude, waveform, and envelope type parameters. To ensure that the latter two could be manipulated independently of amplitude, a separate procedure was used, prior to the main part of Experiment 1, to equalize the stimuli with respect to the subjective intensity of vibration. Ten subjects that did not participate in the main experiment were recruited for this equalization experiment (mean age 23.1 years, STD = 6.1 years, average mass 71.1 kg, STD = 10.4 kg), which was based on a two-alternative forced-choice adaptive staircase method. On each trial, subjects walked across two configurations of the plate differing in vibration feedback type and amplitude and reported whether the first or second vibration felt stronger. The order of the two stimuli was random from trial to trial. One of the two, the standard, was always the high-amplitude white noise stimulus (WC1). The other, the comparison, was one of the remaining eight stimulus types parametrized by waveform and envelope (type SC, SP, SD, WP, WD, TC, TP, or TD). The amplitude of the comparison was controlled by a staircase method that tracked the point of subjective equality, i.e., the amplification factor for the comparison stimulus that rendered it as intense as the standard. If the subject indicated that the comparison felt stronger (respectively weaker), then its amplitude was reduced (respectively increased) by one step unit. The step size was initially large (10 dB) and became smaller (3 dB) after two reversals in the direction of the threshold-tracking sequence. Each staircase was run for 12 reversals, and the point of subjective intensity equivalence was calculated as the average between the last 8 reversals. A total of 16 staircases (8 interleaved pairs) were completed by each subject. Other details were as described under “General Procedure”.

The results of this procedure were used to assign the values of the gain 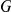 of the Experiment 1 stimuli. The stimuli were regarded as equal in subjective intensity, within limitations determined by experiment duration and inter-subject variability. Table 1 reports the measured peak and RMS gain values for the equalized stimuli, which are labeled with a two-letter string, with the first encoding waveform (S, W, T =  Sinusoidal, White noise, Textured noise), the second encoding envelope (C, P, D =  Constant, Proportional, Dynamic). Values are reported for the high-amplitude (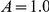) stimuli. Both experiments also included stimuli with 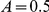. Amplitudes were verified by accelerometer measurement while the plate was loaded by a footstep. For the noise stimuli (W and T), since the absolute peak could vary between presentations, a stable measure was obtained as the median of peak amplitudes on a set of 10 ms windows spanning the highest amplitude interval.

**Table 1:** Experiments 1 and 2: Peak and RMS amplitudes of plate acceleration, 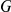 for the high amplitude 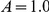 vibration stimuli.

		SC	SP	SD	WC	WP	WD	TC	TP	TD
Exp. 1	Peak ( 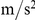 )	2.9	3.7	6.8	17.4	18.4	18.2	3.3	4.6	6.8
	RMS ( 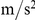 )	1.74	2.4	4.4	10.9	8.5	10.9	1.2	1.75	2.4
Exp. 2	Peak ( 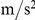 )									0.86
	RMS ( 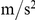 )									0.29

**Stimulus labels:** S, W, T =  Sinusoidal, White noise, Textured noise waveform; C, P, D =  Constant, Proportional, Dynamic envelope.

#### Procedure

For each stimulus presentation during the experiment, subjects walked across the plate and rated its compliance using a slider labeled “most compliant” and “least compliant” at the two extremes. Subjects were informed that they might, at times, feel vibrations via their feet, but no further elaboration was given. The first experimental block was a warm-up period in which subjects tried all configurations of the plate that would be presented. In this period, they were instructed to focus on the maximum and minimum compliance within the stimulus set. During the remainder of the experiment, subjects were asked to use the entire range of the slider when rating the stimuli. The 18 equalized stimuli were presented in blocked randomized order, each stimulus being presented once on each of 12 blocks, for a total of 216 trials. The resulting data consisted of twelve compliance ratings per stimulus from each subject. The entire experiment lasted 90 minutes. There was a pause of two minutes between blocks, and a pause of five minutes after the sixth block. Additionally, there was a pause of at least five seconds between stimuli, as subjects entered their responses. Other details were as described under “General Procedure”.

### Experiment 2

The experiment investigated the extent to which vibration feedback modified perception of the compliance of the floor plate when both vibration amplitude and plate stiffness were manipulated. The resulting data consisted of the proportion of responses in which the comparison was judged more compliant, for each stiffness and amplitude level.

#### Recruitment

Twenty new subjects participated in the experiment (mean age 23.1 years, STD = 4.1 years, average mass 69.8 kg, STD = 11.2 kg). Other details were as described under “General Methods”.

#### Apparatus

**Figure 3:**
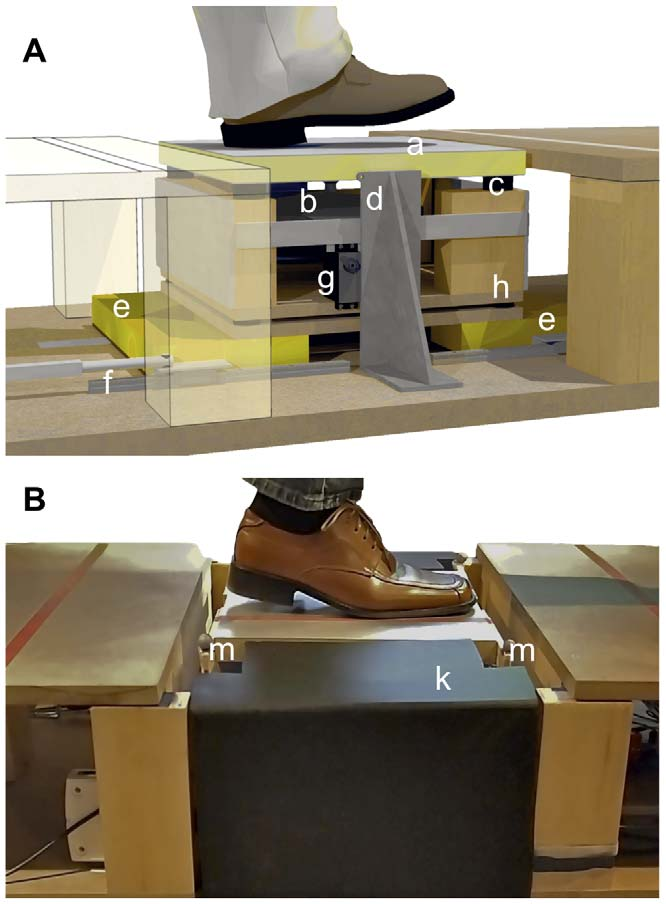
View of the variable compliance mechanism. A. Users stepped onto vibrating plate a, which was driven by vibration actuator b and mounted on suspension c. The plate displaced in the vertical direction, guided by low-noise ball bearing slides d, and compressing a pair of foam inserts e. To produce the commanded compliance, the foam inserts e were positioned by the linear servomechanisms f before each stimulus presentation, while the plate assembly was lifted by servos g. Participant-applied forces were measured by load cells h under four corners of the plate assembly. B. Image of the apparatus and shoe as used in the experiment. Opaque panels k and fabric (not shown) hid the device configuration from subjects' view. Four optical motion capture markers m tracked the displacement of the plate with high precision.

The plate was integrated with a novel mechanism that allowed it to displace vertically with low friction, and that allowed us to vary the mechanical stiffness of the plate precisely for each stimulus within a range from 40 to 160 N/mm. An automated servomechanism was used to change the amount of surface area of a pair of highly recoverable, 3 cm thick foam pads inserted beneath the plate (Fig. 3).

**Figure 4:**
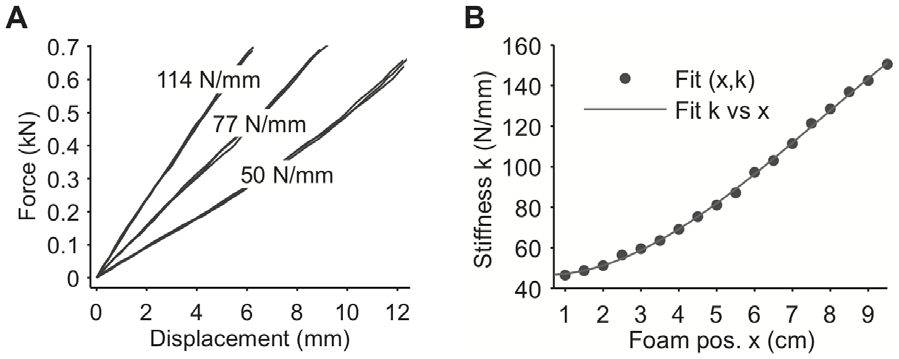
Force-displacement profiles and fits of stiffness vs. foam position used to calibrate the apparatus. A. Examples of three compression profiles are shown (overlaid) for each of three stiffness values. Each calibration was based on sixty such profiles, with more than 3000 data points each. B. Calibration curve fit of stiffness vs. foam position based on measurements at each position and stiffness.

A calibration procedure made it possible to specify plate stiffness, in values of N/mm, via computer control of foam position, with a mean accuracy of about 1%. Force measurements from the load cells in the apparatus were combined with position measurements from a precise motion capture system (OptiTrack, Model FLEX:V100R2). Calibration was performed using least squares regression fit of force to 60 force-displacement profiles consisting of more than 3000 measurements each (Fig. 4). As illustrated, the force-displacement relationship was approximately linear. Measurements from each force-displacement profile were acquired by loading the plate with a typical footstep, since, due to the finite recovery time of the foam pad, the measured stiffness could depend on the temporal profile of the load.

To ensure that the presented stiffness remained accurate, the calibration process was repeated four times in the course of the experiment, between experimental sessions. The minimum stiffness was 52 N/mm, corresponding to an absolute maximum displacement of approximately 2 cm when a subject walked across it with a maximum downward force of about 1000 N. We avoided using softer settings, as we found that it could otherwise become difficult for subjects to step normally and stably across the plate without the need to look down, which was discouraged (see “General Procedure”). The maximum stiffness was 146 N/mm, which was close to the highest level that could be well controlled. At stiffer settings, the intrinsic compliance of the vibration mounts (location b in Fig. 3) would have a non-negligible influence on the plate compliance in ways that depended on foot location and orientation. In addition, stiffness discrimination underfoot would likely have become less reliable [21].

All motorized movements resulting from compliance changes were performed smoothly to minimize any vibrations due to the mechanism that might otherwise provide information about the foam configuration. For the same reason, the duration of any mechanical reconfiguration was kept constant (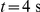).

#### Stimuli

Stimuli were configurations of the floor plate with one of seven different stiffness levels (52, 64, 76, 90, 106, 124 or 146 N/mm) and one of three vibration feedback conditions. The vibrations were the textured, dynamic type (TD). Two different non-zero vibration amplitudes were used, respectively 18 dB and 24 dB lower than the high amplitude (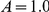) TD stimuli from Experiment 1, as well as a no-vibration condition. These lower amplitudes were selected through pre-testing to ensure that the resulting psychophysical data would be useful. Although Experiment 1 demonstrated that all vibration stimuli could increase perceived compliance, the TD type was selected for further testing because, among those with the highest mean compliance ratings (within one standard error of the mean), they had the shortest duration and one of the lowest RMS amplitudes, limiting the possibility of sensory adaptation during vibration stimulus presentation. The seven stiffnesses and three vibration levels resulted in 21 different comparison stimuli.

#### Procedure

The experiment was based on the psychophysical method of constant stimuli, using a two-alternative forced-choice paradigm. Subjects walked across pairs of configurations, one after the other, and responded indicating which felt more compliant. Vibration was added to the comparison stimulus, except in the “no-vibration” condition, and was never added to the standard. The resulting data consisted of the proportion of responses in which each comparison configuration (that is, stiffness and vibration level) was judged more compliant.

The standard and comparison were presented, as in Experiment 1, in sequential randomized order. Because stiffness was manipulated in the experiment, subjects were required to pause for five seconds between each half of a stimulus pair, in order to provide enough time for the stiffness modification to complete. They were automatically cued to pause and to continue by the software graphical user interface. The timing of this pause was always the same, to avoid giving any indication of the amount of change in compliance. Other details were as described under “General Procedure”.

Subjects were told that during the main experiment they would be asked to respond indicating which of the two configurations felt more compliant. They were told that they might, at times, feel vibrations via their feet, but no further elaboration was given. The first experimental block was a warm-up period in which subjects tried six randomly generated stimulus pairs, consisting of random stiffnesses in the range used in the experiment, and vibration feedback of type WC, with a similar intensity to that used in the main experiment, although the type was different.

During the main experiment, all stimulus pairs were presented in each block, in randomized, balanced order. Randomization of stimulus order was independent for each session and each subject, and no blocks were repeated. Subjects were required to leave the apparatus and pause for one minute between blocks, and for four minutes after each third block. They each completed three experimental sessions, comprising a total of twenty blocks. The duration of the first two sessions was 90 minutes and that of the last session was 1 hour. No more than two experimental sessions, separated by at least two hours, were permitted for any subject on a single day. Each subject was presented with each of the 21 pairs a total of 20 times.

Subjects completed a post-experiment questionnaire and interview, which asked whether the vibrations were felt, and what decision strategy was used (see Results).

#### Psychophysical detection thresholds for the stimuli

Immediately after subjects completed the questionnaire, they participated in a final stage of this experiment, which measured their psychophysical detection thresholds for amplitude. The stimuli consisted of individual configurations of plates, set to the median stiffness level of 90 N/mm, accompanied by vibration feedback of type TD, as used in the main experiment. Amplitude was manipulated independently during the procedure.

The threshold-measurement procedure was based on a single-interval adaptive yes/no staircase method developed by Lecluyse and Meddis for auditory threshold testing [54]. They found, through experiments and simulations, that this procedure yielded similar thresholds to those obtained with two-interval forced choice or maximum likelihood methods, resulted in less variation, and required fewer trials. The latter was a consideration in our experiment, because of the required level of activity and duration, which could lead to fatigue.

Stimuli were presented one at a time, and amplitude was controlled by the staircase procedure. Subjects responded after each presentation indicating whether they felt a vibration from the plate or not. They were instructed to be as sensitive as possible without guessing. When they responded “yes” (resp. “no”), then the amplitude was reduced (resp. increased) by one step unit. The step size was initially large (10 dB) and was reduced to a smaller level (3 dB) after two reversals in direction of the threshold-tracking sequence. For each comparison, one staircase was started at a high amplitude randomly chosen between +8 dB and +15 dB (referenced to the Experiment 2 stimulus amplitude of 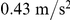) and one at a low amplitude between 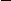
8 dB and 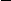
15 dB. The two staircases were interleaved, with one of the two randomly selected for presentation on each trial. In order to prevent guessing, 13% of the trials were randomly selected as catch trials, in which no vibration was present. Subjects were warned that if they answered “yes” on a catch trial, both staircases would be re-started. The median number of times that subjects were caught guessing in the experiment was 1.5 (minimum zero, maximum three). In addition, on every 10th trial, subjects were presented with a no-vibration stimulus and were told that no vibration was present, to remind them how that condition felt.

Each staircase was continued until 12 reversals were reached. A total of six staircases (three interleaved pairs) were completed by each subject, and the threshold was calculated as the average between the last eight reversals from all six staircases. Subjects were required to pause for 2 minutes between staircase pairs. The total duration for each subject was approximately 30 minutes.

## Results

### Vibration Stimulus Factors Influencing Subjective Compliance Judgements

**Figure 5:**
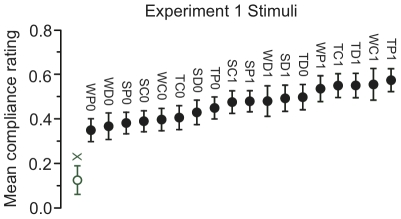
Experiment 1: Subjective compliance ratings for all 19 stimuli, averaged across subjects. A higher value means more compliant (less stiff). “X” labels the no-vibration stimulus. Others are labeled with a 3-letter string encoding waveform, envelope, and amplitude level (0, 1 =  Linear amplitude 0.5, 1.0). Error bars  =  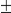
1 standard error of the mean (SEM). All vibrating stimuli were significantly more compliant than the no-vibration stimulus.

Data from Experiment 1 were analyzed to determine the effect of vibration feedback type on compliance ratings. Mean ratings for all the 19 stimuli are shown in Figure 5. Paired t-tests showed that all 18 vibrating stimuli were perceived as significantly more compliant than the non-vibrating one (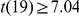, 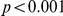).

**Figure 6:**
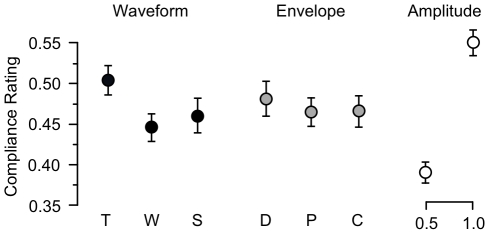
Experiment 1: Average compliance ratings for the three stimulus factors “waveform type”, “envelope type”, and “amplitude”. Stimulus labels are as given in Fig. 5. Error bars: 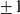 SEM.

We further analyzed compliance ratings in Experiment 1 with a within-subject repeated measures ANOVA, with amplitude, envelope, and waveform as factors. Mean compliance ratings for the values of each factor are shown in Fig. 6. The one-way effect of amplitude was significant (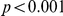, 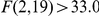, 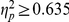), as was that of waveform (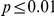, 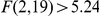, 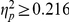), but the effect of envelope type was not significant (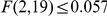, 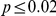, 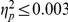). There were significant effects of all two-way interactions, amplitude 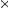 waveform (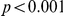, 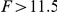, 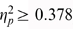), amplitude 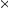 envelope (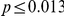, 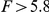, 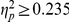), and waveform 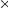 envelope (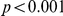, 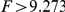, 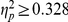). There was no significant three-way effect (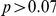, 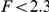, 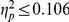). Based on this analysis, amplitude had the largest influence on compliance judgments, while the effects of waveform and of all two-way interactions were smaller.

### Effect of Vibration Feedback on Compliance Perception

**Figure 7:**
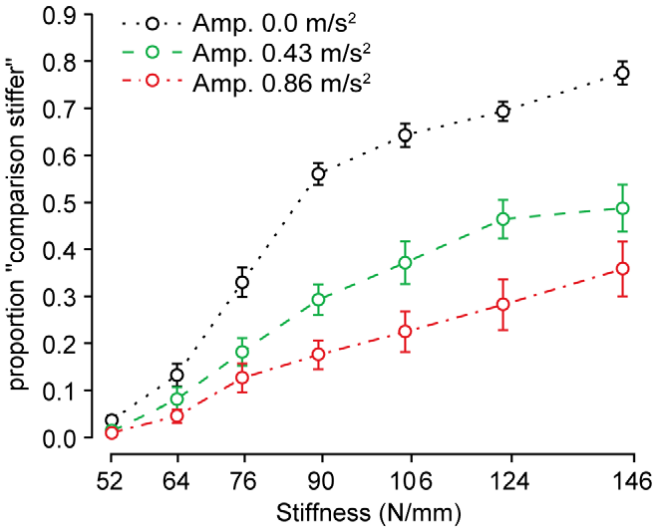
Experiment 2: Mean proportion of comparison stimuli judged stiffer than the standard. A higher proportion implies a judgment of “stiffer”. The standard had a stiffness of 90 N/mm, as indicated by the dashed line, and did not present any vibration feedback. Results shown are averaged between all 20 subjects. Error bars: 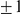 SEM.

The results of Experiment 2 consisted of proportions of responses at which the standard stimulus, a non-vibrating plate with stiffness 90 N/mm, was judged less stiff than a comparison that varied in stiffness and in vibration amplitude. Figure 7 presents the average response proportions. A one-way ANOVA of the response proportions for the factor *amplitude* indicated that vibration significantly decreased stiffness at each stiffness level (see Table 3). Although the variation in responses was larger at higher stiffnesses, the effect of amplitude on subjective compliance was proportionally larger, yielding a higher level of significance. For all subjects, average response proportions at the largest amplitude level (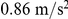) were higher than in the no-vibration case.

**Figure 8:**
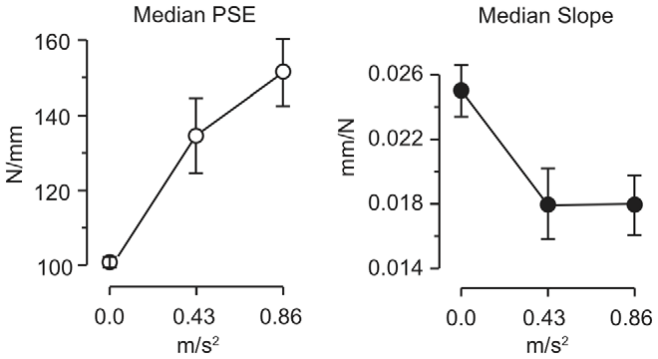
Experiment 2: Median values of the PSE and slope from per-subject psychometric fits at each vibration amplitude level. Error bars: 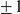 SEM (outliers excluded).

Binary response data at each vibration amplitude level from each of the 20 subjects were fitted to a cumulative normal distribution using a probit regression model. A total of 60 fits were performed. The models explain 86.5% of the variance in the data with Pearson correlation 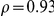. The slope, intercept, and point of subjective equivalence (PSE) in stiffness were computed from each fit. Median values in each condition are given in Table 2 and shown in Figure 8. We investigated influences of vibration amplitude on the fit parameters using a nonparametric Friedman test, in order to ensure that the analysis would remain robust to outliers in the data. The latter resulted from a few subjects whose response proportions increased slowly with stiffness in the high-amplitude condition, leading to unusually small slope values, and large PSEs. Vibration amplitude did not significantly affect intercept (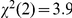, 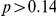), indicating that subjects were not biased to indiscriminately answer “softer” when vibration amplitude was higher. PSE increased significantly with amplitude (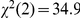, 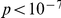), indicating that the stimulus was perceived as softer when vibration was present, and slope also increased (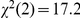, 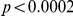), indicating that stiffness discrimination performance was impaired in the presence of vibration. Nonparametric repeated-measures tests contrasting all three amplitude levels indicated no significant effect on intercept (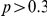, Bonferroni corrected, BC), but did reveal an effect of amplitude on PSE for all pairings (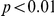, BC). There was a significant effect on slope between the no-vibration and either high- or low-amplitude conditions (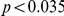, BC), but not between the low- and high-amplitude vibration conditions (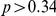, BC). Increasing the vibration amplitude level from low to high thus increased the bias in the PSE for stiffness estimation without further decreasing discriminability.

**Table 2:** Experiment 2: Mean proportions of comparison stimuli that were judged to be stiffer than the standard, as a function of stiffness, with different levels of vibration.

	Response Proportion at Amp. 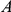			one-way ANOVA	
Stiffness	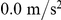	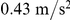	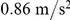	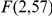	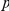
52 N/mm	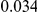	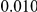	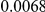	4.3	0.018
64 —	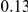	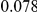	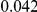	4.24	0.019
76 —	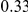	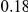	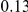	12.5	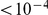
90 —	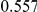	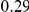	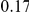	50.3	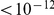
106 —	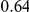	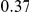	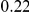	31.6	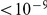
124 —	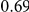	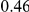	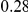	26.9	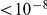
146 —	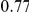	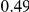	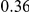	22.0	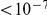

**Table 3:** Experiment 2: Median values of the PSE, slope, and intercept from per-subject psychometric curve fits at each vibration level.

	Median Value at Peak Amplitude 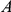			Friedman Test	
	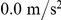	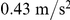	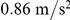	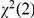	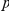
PSE (N/mm)	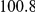	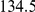	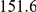	34.9	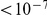
Slope (mm/N)	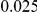	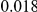	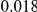	17.2	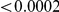
Intercept	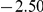	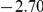	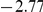	3.9	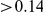

**Figure 9:**
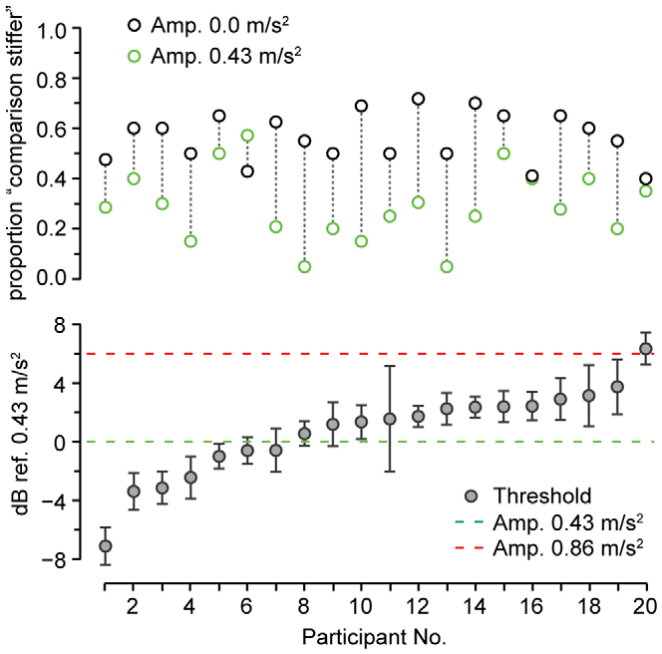
Experiment 2: Psychophysical amplitude detection thresholds and response proportions at stiffness level 90 N/mm. Top: Response proportions for all 20 subjects at two lowest vibration levels, and stiffness level 90 N/mm. Bottom: Amplitude threshold levels, displayed in dB referenced to 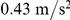. Subjects are sorted in order of increasing threshold (same ordering top and bottom). The dashed lines indicate the amplitudes of the vibrating stimuli used in Experiment 2. Error bars: 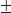
2 SEM.

Experiment 2 also measured psychophysical amplitude thresholds for detection of the vibration stimuli, for the same subject pool used in the main part of the experiment, with the plate stiffness set to the median value of 90 N/mm. These thresholds were measured in shoes, and would likely be lower if direct skin contact were involved. The measurements were based on a fast, single interval yes-no procedure with catch trials [54]. Although Lecluyse and Meddis found this method to yield similar thresholds to those obtained using a two-alternative forced choice task, it could be argued to have led some subjects in our experiment to adopt conservative criteria for responding during the detection staircase, which would yield an overestimate of the thresholds. Figure 9 presents the results of the measurements. The mean threshold was 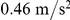, with standard deviation 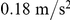. Measured thresholds for 10 of the 20 subjects were higher than the low-amplitude (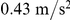) stimulus by more than two standard errors of the mean. For the subgroup of 10 participants with the highest thresholds, we analyzed proportions of responses “more compliant” from the no-vibration (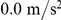) to the low-amplitude (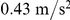) vibration condition at the same stiffness value (90 N/mm) as was used in the threshold measurement. A paired two-tailed t-test revealed a significant effect of amplitude on these response proportions (mean 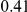 vs. 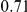 with 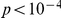, 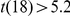); see Figure 9. The median PSEs of the psychometric fits for the same subgroup of 10 participants were also significantly higher in the low-amplitude condition than in the no-vibration condition (median 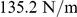 vs. 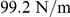, Friedman 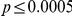, 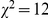), and were close in value to the median PSEs for the complete subject pool. However, the threshold values for the entire subject pool (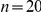) were not significantly correlated with PSE values in either the low or high vibration amplitude condition, with differences in PSE values between vibration and no-vibration cases, or with mean response proportions at stiffness 90 N/mm (Spearman 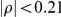, 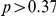 in all cases).

The post-experiment questionnaire that was completed by Experiment 2 subjects prior to the threshold measurement included the question: “Did you feel any vibration produced by the tile?” Five out of twenty subjects responded “No”. A sixth noted that he felt vibration on just 5% of trials, and a seventh reported feeling no vibrations, but did report feeling “a creaking”, “like stepping on an old hardwood floor” on some trials. The remaining 13 subjects answered “Yes”.

## Discussion

These experiments demonstrate that the perceived haptic compliance of a walking surface is increased in the presence of plantar cutaneous vibration feedback. In Experiment 1, we found that the largest effect on perceived compliance was due to vibration amplitude, and that other stimulus factors had a weaker influence. We also found that an increase in perceived compliance could be achieved with types of vibration feedback that differed in waveform, amplitude envelope, or the frequency distribution of their energy.

In Experiment 2, we found that vibromechanical stimuli with peak amplitudes of only 0.43 and 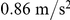 could elicit an increase in perceived compliance. These levels were 18 dB smaller than those used in Experiment 1. This held at all tested levels of stiffness. A substantial increase in the stiffness of the vibration-augmented plate was required for it to be perceived as having the same stiffness as the non-augmented one, as the vibration feedback produced positive relative shifts in the median PSE values for stiffness of 34% and 50% at the two amplitude levels.

The amplitudes used in Experiment 1 were comparable to those that are experienced during normal walking on natural granular materials such as sand or gravel [31], [36], while those used in Experiment 2 were significantly weaker. Through pre-testing for Experiment 2, we determined that higher amplitudes tended to dominate the influence of mechanical stiffness over the range explored. The upper limit of the stiffness range used approached a level at which stiffness perception underfoot is less reliable [21], while the smallest stiffness was near the limit of what we determined subjects could comfortably and safely walk on with this apparatus.

None of the experiments involved training, and the effects observed did not require awareness that vibration feedback was being provided. We can conclude that vibration felt during stepping on a rigid surface is combined with the mechanical stiffness of the surface in the haptic perception of compliance. In addition, the results show that the variation of vibration feedback alone is sufficient to elicit a percept of compliance.

The compliance estimation task adopted in this study resembled prior experiments in which subjects used their hands or arms to estimate the haptic compliance of spring-loaded mechanisms or other objects with non-deformable surfaces [17], [19], [22]. Based on those results, and on considerations of contact mechanics, it was expected that subjects in our experiments required both force and displacement information (from kinesthetic and tactile channels) in order to judge compliance. In this light, it appears that added vibration feedback results in a modification of force and/or displacement information that increases compliance estimates. As noted earlier, localized vibration stimulation of the foot sole has been shown to have a similar effect on postural control to an increase in force sensation at the same location [43], [44], and this could be thought to influence compliance judgments. However, an ideal observer combining force 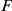 and displacement 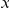 to estimate compliance using the formula 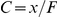 would produce lower compliance estimates as force sensation is increased. Our results show an opposite tendency, so it appears unlikely that the observed effects on perceived compliance were mediated by increased sensations of applied force. Furthermore, stimuli used in Experiment 2 had a peak amplitude of 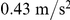, less than 0.5% as large as the smallest amplitude used in the aforementioned studies.(To compare stimuli, we computed accelerations used in experiments by Roll et al. [43], [44] from the stimulus properties they reported.) Furthermore, in our experiment, stimuli were felt through a shoe, whereas those used in the aforementioned studies involved direct skin contact.

It might be suggested that the observed results could be attributable to sensory adaptation of SA I afferents due to the vibromechanical stimuli, which could yield a reduction of force estimates. However, in our experiments, exposure times averaged less than 1 second, with at least 5 seconds between presentations, whereas mean adaptation times for SA I afferents are about 10 seconds [55], [56]. Additionally, Experiment 2 was based on pairwise comparison of two stimuli, only one of which could include vibrations. Also, the low-amplitude Experiment 2 stimuli were 0.6 dB weaker than the mean psychophysical detection threshold measured for our subject pool. While this might be partly attributable to a tendency of the measurement method used to overestimate the thresholds, it nonetheless appears unlikely that these stimuli could have produced a significant adaptation of SA I responses, even after long exposure times. Furthermore, no subjects reported feeling any desensitization in their feet, and several were unaware of the vibrations. Thus, it appears unlikely that sensory adaptation played a significant role.

Conversely, prior studies have demonstrated that sub-threshold levels of plantar stimulation with vibration noise can enhance cutaneous sensitivity in the foot soles, stabilizing posture [57]. Although such an effect, if present, could have improved haptic force discrimination in our experiment, it would not be expected to influence mean compliance estimates, so does not seem to be able to explain our main results.

Experiments 1 and 2 compared the perceived compliance of plates with and without vibration feedback. A priori, due to this categorical difference, subjects could have responded based on cognitive criteria unrelated to a sensation of compliance. However, there are several reasons we do not believe cognitive effects played an important role. Subjects were consistent in responding that the vibrating plates were more compliant, and no subject inverted this relation. Experiment 2 results did not indicate any tendency on the part of subjects to respond indiscriminately that the vibrating stimulus was “softer” independent of actual compliance. In addition, vibration had a significant influence on compliance at both near-threshold levels (Experiment 2) and at much higher ones (Experiment 1). Furthermore, some subjects in Experiment 2 reported that they were not consciously aware of the presence of vibration feedback. Finally, a few subjects described what they felt in a way that is consistent with the notion of a material being compressed underfoot, and similar responses have been received over the course of numerous demonstrations of the apparatus to naive users.

Taken together, our findings appear to be consistent with the hypothesis that vibration feedback supplied a cue that tended to increase perceived displacement during stepping, due to a sensorimotor contingency similar to that experienced when stepping on a natural material (e.g., snow, gravel) or displacing a mechanism with friction (e.g., a pedal or slider). Assuming this to be the case, and supposing that perceived force was not affected, an ideal observer would infer an increase in compliance that grows linearly with the increased sensation of displacement, due to the relation 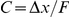. In this model, a relative increase in estimated displacement of 25.0% and 33.5% in the low- and high-amplitude vibration conditions, respectively, would be required to explain the shifts in median stiffness PSE values measured in Experiment 2.

One counterintuitive finding is that stimuli with amplitude envelopes that were constant could evoke an increased sensation of compliance, contrary to the idea that vibration supplies a force-dependent displacement cue. However in the conditions of this study, vibromechanical energy transmitted to the leg increased with applied force 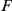, due to the increased coupling of foot and plate, even for constant stimuli. As a result, the feedback could appear to have been generated by a stepping action even when there was no explicit relation with applied force, due to the transitive nature of foot-plate contact.

A number of disorders, the most common being diabetes, can impair cutaneous tactile sensation in the feet and have detrimental effects on locomotion [8]–[10]. This has led various researchers to investigate relations between sensory impairment and control of balance or locomotion. The present study suggests that vibrotactile sensation may be more involved in the regulation of walking in natural environments than has been acknowledged. One pilot study found that step-synchronized plantar vibration feedback during foot-ground contact may improve locomotion in Parkinson's disease patients, but there were insufficient controls to rule out learning or attentional effects [58]. However, it is plausible that the effects investigated here could play a role in such settings.

Through preliminary body kinematic analyses using motion capture measurements we have found indications of postural modifications during stepping when plantar vibration feedback is supplied. This would be consistent with postural kinesthetic illusions that, absent restraints, result in compensatory body sway [44]. If confirmed, such results might one day prove useful for the development of gait rehabilitation techniques or vibrotactile orthotics. We intend to explore these questions further in future work. Finally, we note that the same compliance illusion seems to be present during interaction via the hands. This is also something we plan to investigate further, in order to situate our results relative to prior literature on manual haptic perception.
